# Lack of association of ovariectomy-induced obesity with overeating and the reduction of physical activities

**DOI:** 10.1016/j.bbrep.2019.100671

**Published:** 2019-08-08

**Authors:** Eiji Nishio, Takanori Hayashi, Masashi Nakatani, Noriko Aida, Risa Suda, Takuma Fujii, Toru Wakatsuki, Shinichiro Honda, Nobuhiro Harada, Yohei Shimono

**Affiliations:** a Department of Obstetrics and Gynecology, Fujita Health University School of Medicine.; b Department of Biochemistry, Fujita Health University School of Medicine.; c Division for Therapies against Intractable Diseases, Institute for Comprehensive Medical Science (ICMS), Fujita Health University.; d Department of Health Science, Fujita Health University School of Medicine.; e Department of Biochemistry, Faculty of Pharmaceutical Sciences, Fukuoka University.

**Keywords:** Obesity, Estrogen, Postmenopausal women, Exercise

## Abstract

Obesity commonly occurs in postmenopausal women, increasing the risk of various diseases. Estrogen can prevent obesity by activating lipid metabolism and suppressing depressive behavior. However, the reasons for obesity in postmenopausal women are not clearly elucidated.

To mimic the effect of estrogen decline in postmenopausal women, we analyzed the behavior and the lipid metabolism-related genes, PPARγ and CD36 in ovariectomized (OVX) mice. The OVX mice showed increased visceral fat mass and PPARγ and CD36 expression in the visceral fat. In contrast, they were not significantly affected in terms of physical activity and food intake. Further, subcutaneous supplementation of estrogen effectively suppressed the increase in subcutaneous and visceral fat mass in OVX mice.

We conclude that obesity in postmenopausal women is unlikely to be caused by overeating and reduction in physical activity, and subcutaneous supplementation of estrogen is an effective strategy to prevent obesity in postmenopausal women.

## Highlights

• We studied the effects of estrogen deficiency/supplementation on obesity in mice.
• After ovariectomy, the study animals received transdermal injections of estrogen.
• Exogenous 17β-estradiol appears to mediate the effects of menopause on obesity.
• Obesity in the mice was caused solely by estrogen deficiency, not overeating.
• Estrogen deficiency altered the mechanics of fat deposition in the mice.

## 1 Introduction

Obesity, defined as an excess of body fat, is a growing public health crisis associated with the development of many disorders, including type 2 diabetes and cardiovascular disease [1,2] Body fat is distributed in subcutaneous adipose tissue and visceral fat tissue, which have different metabolic properties. The storage of body fat, resulting in weight gain, is thought to be due to overeating and physical inactivity. Menopause causes deterioration of lipid metabolism and increases the tendency to develop obesity [3,4]. Ovariectomized (OVX) mice have been used to study changes in postmenopausal metabolism and reported to gain weight due to changes in lipid metabolism, as do menopausal women [[5], [6], [7]].

Aromatase (the cyp19 gene product) is a key enzyme in the biosynthesis of estrogen from androgen. Previously, we investigated the effect of estrogen on exercise and behavior using Aromatase Knockout (ArKO) mice. ArKO mice showed increased body weight compared to wild-type mice, but this effect decreased over time [8]. Weight gain in OVX mice may be caused by abnormal lipid metabolism but also by decreased momentum, as in the case of ArKO mice.

The relationship between obesity and menopause appears to be mediated by exogenous 17β-estradiol (E2) in a way that shows racial disparities, although the interconnected pathways are not fully characterized [9,10]. A few studies have evaluated the effects of estrogen deficiency on weight gain. However, it is not clear how lack of exercise or overeating is related to menopause. To explore this question further, we used an experimental animal model.

## 2 Materials and methods

### 2.1 Animals

Five-week-old female C57BL/6 mice were purchased from Japan SLC, Inc. (Shizuoka, Japan). They were housed in polycarbonate cages with wood pulp bedding, which was changed once a week. Mice received a pellet rodent diet (CE-2, CLEA Japan, Inc., Tokyo, Japan) and tap water. Mice were singly housed under a controlled photoperiod with a 12:12-h light-dark cycle and temperature (22–24 °C) throughout the experimental period. Female mice underwent ovariectomy at 8 weeks. Animal care and experiments were conducted in accordance with Fujita Health University guidelines.

### 2.2 Body weight and ratio of body fat

Body weight and dietary intake from 8 to 20 weeks of age were compared between the sham control (SHAM) and ovariectomized (OVX) mice. The percentages of subcutaneous and visceral fat were assessed by computed tomography in 20-week-old wild-type (WT) and OVX mice.

### 2.3 Locomotive behavior

Locomotive (or exploratory) behavior was observed in open field cages to measure travel distance and resting time. All behavioral tests were conducted during the dark phase of the light/dark cycle, starting 2 h after lights were switched off. The locomotive behavior was video-recorded in an open field cage (53 cm long × 35 cm wide × 30 cm high) with an infrared night scope for 15–30 min per session and analyzed on the basis of travel distance, resting time, and parallelism index using the behavior analysis software SMART (Panlab, S. L., Barcelona, Spain). Travel distance and resting time during a 15-min open field test were analyzed from video-recorded sessions of sham-operated (SHAM) and OVX mice (n = 8) aged 14–16 weeks. The parallelism index, a characteristic feature of locomotor or exploratory movement, was calculated from video-recorded locomotor activities. The index value was calculated using a score of +1 when the movement was straight ahead, and 0 and −1 when the test mouse moved to either side and backward, respectively.

### 2.4 mRNA expression levels of genes associated with obesity or energy metabolism

RNA was extracted from the livers of 20-week-old SHAM and OVX mice, and the mRNA expression levels of genes associated with obesity or energy metabolism were determined by real-time PCR after comprehensive gene-microarray analysis. We quantified the mRNA of peroxisome proliferator activated receptor γ (PPARγ) and cluster of differentiation 36 (CD36). Total RNA was extracted from treated cells using TRIzol reagent (Qiagen, Hilden, Germany) and reverse transcribed using PrimeScript RT reagent kit (TaKaRa Sake USA, Torrance, CA, USA). Quantitative PCR (qPCR) was performed in triplicate using the ABI Perkin-Elmer Prism 7300HT Sequence detection system (Applied Biosystems, Foster City, Ca, USA). Taqman gene expression assays (Applied Biosystems) were used to detect the expression of PPARγ (Taqman Accession ID Mm00440940_m1) and CD36 (Mm00432403_m1); GAPDH (Mm99999915_g1) was used as a housekeeping gene. Relative quantities were determined using the ΔΔCt method, according to the manufacturer's instructions.

## 3 Results

### 3.1 Weight gain and increase of fat in OVX mice

**Fig. 1:**
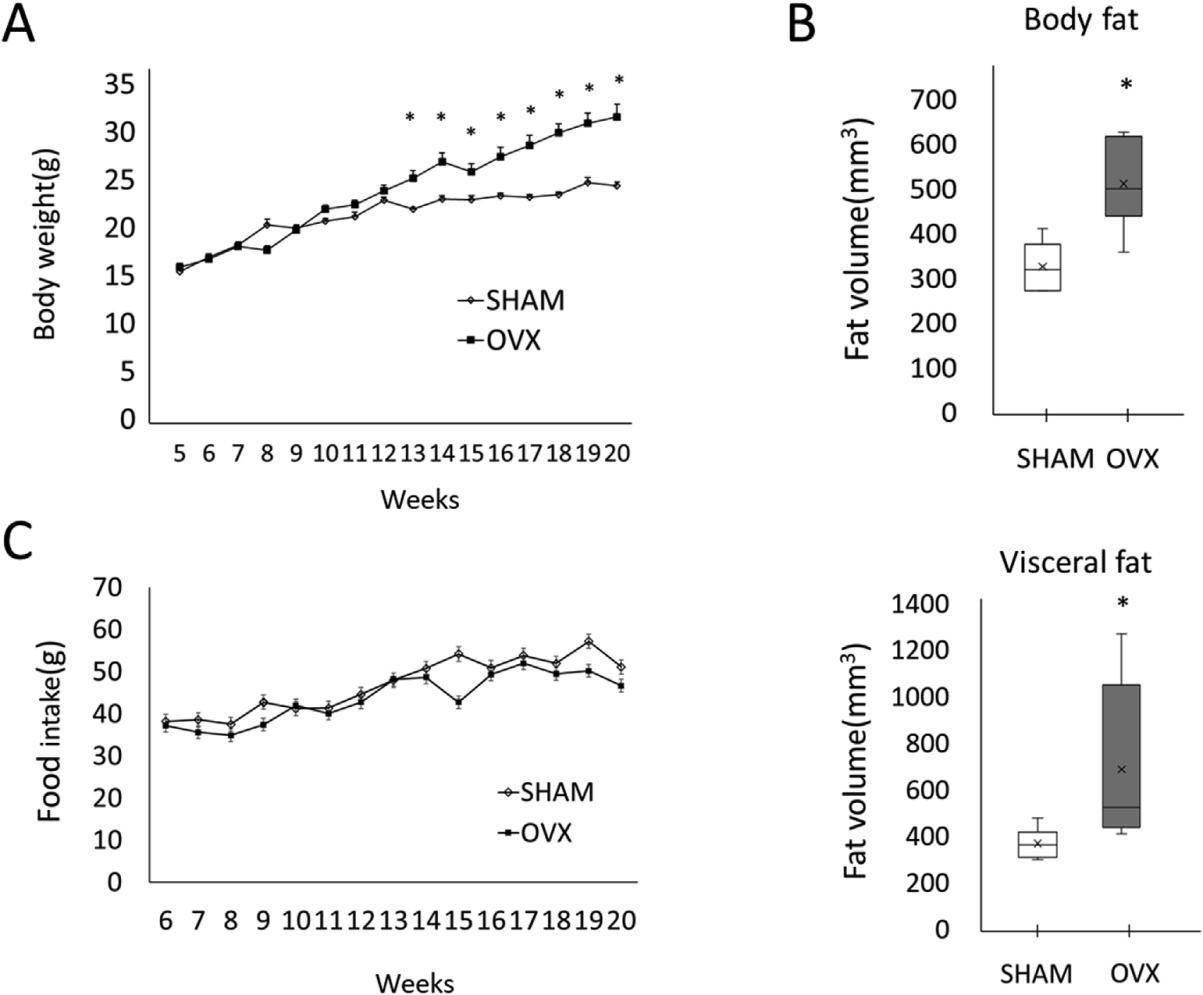
Increase of body fat in OVX mice. A: The body weight of the OVX group was significantly increased compared to that of the SHAM (sham-operated control) group after 13 weeks. The weights of the experimental animals were measured weekly. B: Increase of the body fat and visceral fat in OVX mice. The amount of fat was measured using three-dimensional (3D) images. C: The amount of diet in OVX. The values are expressed as the means ± SD (n = 8). The bar with the asterisk (*) is significant in relation to the SHAM group (p < 0.05).

The weights of experimental animals were measured at weekly intervals. The body weight of the OVX group was significantly higher than that of the SHAM group after 13 weeks (Fig. 1A). Three-dimensional images of fat layers in the OVX group showed that the subcutaneous fat and visceral fat were increased in comparison with those in the SHAM group. The volume of subcutaneous fat was significantly higher in the OVX group (SHAM: 324.1 ± 54.6 mm^3^, OVX: 508.6 ± 99.8 mm^3^, p = 0.0317), and that of visceral fat was also significantly higher in the OVX group (SHAM: 364.7 ± 64.2 mm^3^, OVX: 647.0 ± 341.6 mm^3^, p = 0.0177). The OVX mice received the same amount and type of food as the SHAM mice (Fig. 1C).

## 4 Results for locomotive (exploratory) behavior

**Fig. 2:**
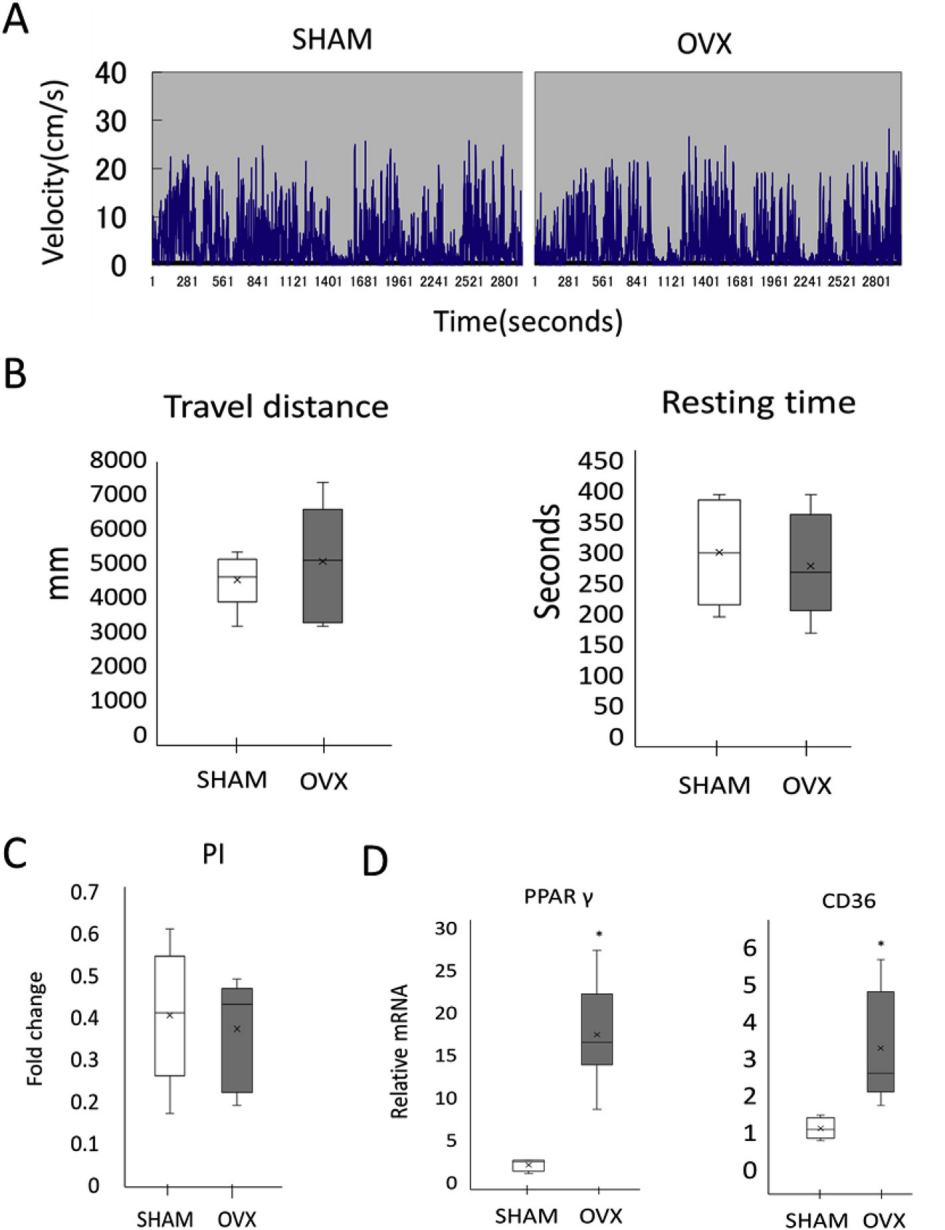
The locomotive behaviors of wild-type and OVX mice. A: Representative results of locomotive activities of mice aged 15 weeks in open field tests. B: The travel distance and resting time during a 15-min open field test. Locomotor activities of SHAM-type (n = 8) and OVX (n = 8) mice aged 14–16 weeks were video-recorded. C: The parallelism index (PI), a characteristic feature of locomotor or exploratory movement, was calculated from video-recorded locomotor activities. D: Relative changes in PPARγ and CD36 mRNA. The values are expressed as the means ± SD (n = 8). The bar with the asterisk (*) is significant in relation to the OVX group (p < 0.05).

No difference in the travel distance was observed between the two groups, as shown in Fig. 2A (SHAM: 4474 ± 816 mm, OVX: 5010 ± 1645 mm, p = 0.429). No difference in resting time was observed between the two groups, as shown in Fig. 2B (SHAM: 298.4 ± 86.6 s, OVX: 276.2 ± 83.4 s, p = 0.647). The parallelism index, another parameter of locomotor activity, indicated the characteristic features of the animals’ movement in the open field. No difference in the parallelism index were observed between the two groups (Fig. 2C). Suppression of weight gain by subcutaneous supplementation of estrogen. The expression levels of PPARγ and CD36 mRNA were significantly higher in the fat of OVX mice than those in control mice (Fig. 2D).

### 4.1 The influence of estradiol

**Fig. 3:**
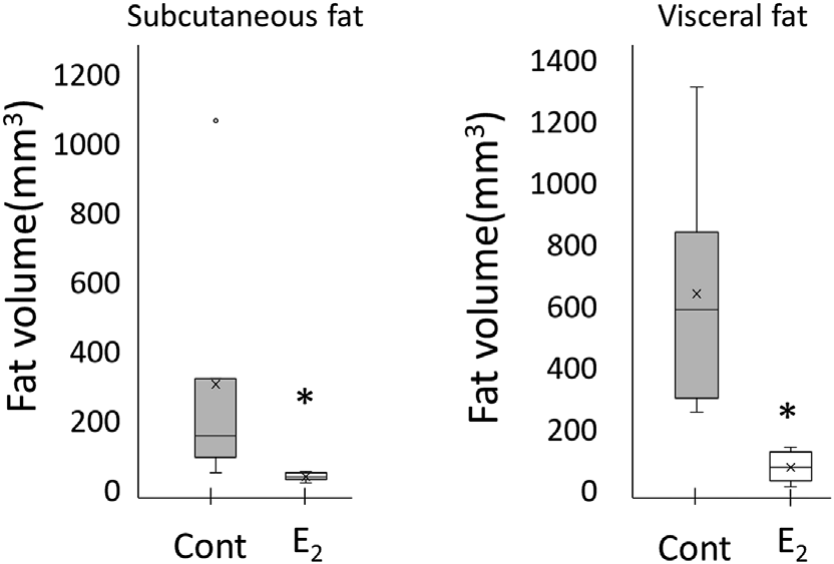
Reduction of subcutaneous fat and visceral fat by percutaneous estrogen supplementation.

The volume of subcutaneous fat was significantly lower in mice that received estradiol via transdermal administration than in those that received a placebo (Fig. 3; placebo: 302.2 ± 91.4 mm^3^, estradiol: 35.9 ± 11.3 mm^3^, p = 0.001). The volume of visceral fat was significantly lower in the mice that received estradiol via transdermal administration than in mice that received a placebo (Fig. 3, placebo: 638 ± 359.3 mm^3^, estradiol: 74.3 ± 47.1 mm^3^, p = 0.0001).

Fat volumes were decreased by percutaneous estrogen supplementation. The values were expressed as the means ± SD (n = 8). The bar with asterisk (*) is significant in relation to E2(+) group (p < 0.05).

## 5 Discussion

Many factors have been associated with obesity. It is generally accepted that perimenopause is associated with increased visceral fat [11,12]. Weight gain is thought to occur due to overeating and physical inactivity [13]. We expected that ovariectomy would increase dietary intake and reduce physical activity, but no differences in dietary intake nor physical activity were observed between the two groups.

Aromatase (the product of the cyp19 gene) is a key enzyme in the biosynthesis of estrogen from androgen. In our previous study, we reported that estrogens locally synthesized in the specific neurons of the perinatal mouse brain directly act on the neurons and play crucial roles in the organization of neuronal networks participating in the control of sexual, aggressive, and locomotive (exploratory) behaviors [7]. We speculate that physical activity depends on the presence or absence of estrogen in the brain. It is assumed that a deficiency in estrogen is by itself capable of causing obesity. If the time that elapses before menopause is short, the chance of experiencing this deficiency is low. As we removed the ovaries of very young (8-week-old) mice in the present experiment, the period during which the mice had fertile cycles was short and they did not experience estrogen deficiency. Estrogen synthesized locally in nerves and fats is thought to affect physiological behavior separately from estrogen generated in the ovaries.

Metabolic syndrome is recognized clinically as the association of central obesity, dyslipidemia, and hypertension. Estradiol is known to decrease the expression of PPARγ [14]. PPAR is a member of the ligand-activated nuclear receptor superfamily and plays a role in mediating adipogenesis and lipid metabolism [15,16]. CD36 is a member of the class B scavenger receptor family and has the ability to bind to oxidized low-density lipoprotein [17]. CD36 is highly expressed in the liver, which has a high capacity for free fatty acid uptake and plays an important role in lipid metabolism. Increased levels of hepatic CD36 expression can increase fatty acid uptake, and triglyceride accumulation [18].

In conclusion, estrogen replacement therapy may prevent the development of obesity due to low estrogen. Zoth et al. reported that estrogen replacement therapy in combination with physical activity could be a very effective strategy to prevent the development of metabolic syndrome induced by overnutrition. Previous studies reported that the effect of postmenopausal hormone therapy on cardiovascular disease may be explained by its effect on body composition, in particular abdominal fat [3,19]. In a 2018 cohort study, menopausal hormone therapy (MHT) was associated with reduced total and visceral adiposity [20]. However, administration of MHT over a long period placed women at increased risk for cardiovascular disease [12,15,16].

The influence of lipid metabolism may vary according to the route of administration. The incidence of vein thromboembolism, such as deep venous thrombosis or pulmonary embolism, increases in people whose body mass index (BMI) is above 25, and it increases more when people are taking both CEE and medroxy progesterone acetate than when they are using CEE alone. Regarding the relationship between MHT and antiobesity agents, there are no reports describing the relationship between transdermal estradiol administration and prevention of obesity. In the present study, we showed that transdermal estradiol injections have the potential to prevent obesity more safely than do oral agents. It is thus likely that coadministration of estrogen through conventional methods and non–oral estrogen therapy can prevent obesity in menopausal women.

## Conflicts of interest

The authors declare no conflicts of interest involving this article.

## Appendix A Supplementary data

Data Profile Data Profile
